# Evaluation of the analytical performance of three chemiluminescence serological assays for detecting anti-SARS-CoV-2 antibodies

**DOI:** 10.1007/s10238-022-00918-w

**Published:** 2022-10-19

**Authors:** Bruna Lo Sasso, Luisa Agnello, Rosaria Vincenza Giglio, Concetta Scazzone, Davide Massa, Anna Maria Ciaccio, Caterina Maria Gambino, Matteo Vidali, Marcello Ciaccio

**Affiliations:** 1 Department of Biomedicine, Neurosciences and Advanced Diagnostics, Institute of Clinical Biochemistry, Clinical Molecular Medicine and Clinical Laboratory Medicine, University of Palermo, Via del Vespro, 129, CAP 90127 Palermo, Sicily, Italy; 2 Department of Laboratory Medicine, Azienda Ospedaliera Universitaria Policlinico “P. Giaccone”, 90127 Palermo, Italy; 3 Unit of Clinical Biochemistry, University of Palermo, 90127 Palermo, Italy; 4 Foundation IRCCS Ca’ Granda Ospedale Maggiore Policlinico, 20122 Milan, Italy

**Keywords:** SARS-CoV-2 antibody, Vaccine, Chemiluminescence, COVID-19, Immunoassay, Anti-RBD IgG

## Abstract

The serology surveillance of SARS-CoV-2 antibodies represents a useful tool for monitoring protective immunity in the population. We compared the performance of three SARS-CoV-2 antibody serological immunoassays in 600 vaccinated subjects after the BNT162b2 mRNA COVID-19 vaccine. All serum samples were evaluated by three different immunoassays for detecting anti-SARS-COV-2 antibodies. All SARS-CoV-2 antibody serological immunoassays could detect, when present, a post-vaccine humoral immune response. Median (interquartile range, IQR) anti-S-RBD IgG, Access SARS-CoV-2 IgG (1st IS) and Access SARS-CoV-2 IgG II levels of the subjects investigated were, respectively, 687 BAU/mL (131–2325), 419 IU/mL (58–1091) and 104 AU/mL (14–274). By studying a cohort of unvaccinated subjects, without previous COVID-19 infection, we found a high specificity for all methods. A high correlation was found between IgG titres. Considering the kinetics of subjects with multiple doses, we observed that percentage decreasing gradients were comparable across methods. Our results suggest that all the SARS-CoV-2 antibody serological immunoassays evaluated in this study are suitable for monitoring IgG titers over time. This study contributes to a better understanding of antibody response in vaccinated subjects using some currently available assays.

## Introduction

The severe acute respiratory syndrome coronavirus 2 (SARS-CoV-2) infection represents one of the most serious global health threats due to its severity and rapid spread worldwide. Although the knowledge of the pathophysiological mechanisms underlying the infection and the related disease (COVID-19) has grown rapidly, many questions are still unsolved, such as the potential long-term sequelae [1–4].

Since the beginning of the pandemic, several vaccines have been developed to confer immunity against SARS-CoV-2. Vaccine-induced immunity consists of T cells and B cells activation leading to the production of antibodies directed against SARS-CoV-2 epitopes. Specifically, all SARS-CoV-2 vaccines are designed to elicit an immune response against the spike (S) protein that is required for the virus binding, fusion, and cell entry.

The evaluation of anti-SARS-CoV-2 antibody levels is helpful for assessing individual protection against SARS-CoV-2 infection [5–12]. Thus, manufacturers have invested heavily in this sector by developing and marketing in a very short time numerous assays with highly different characteristics in terms of analytical method and antibodies detected. The latter can be differentiated according to immunoglobulin class, i.e., IgG, IgM and IgA or total Ig, including all classes, and the recognized epitopes, which can be the S protein, as whole or a fragment, and nucleocapsid (N). Among all, the IgG directed against S and, especially, toward the receptor-binding domain (RBD) of the S1 subunit of the S, provides the most informative value on the individual immunological protection against SARS-CoV-2 infection because they mask the virus binding sites to the ACE2 receptors of the target cells.

The analytical methods for measuring antibody levels can be based on enzyme-linked immunosorbent assays (ELISA), chemiluminescence immunoassays (CLIA), lateral flow immunoassay (LFIA) and fluorescent microparticle immunoassay (FMI). Among these, ELISA and CLIA are the most used in clinical practice [13, 14]. CLIA has several advantages over ELISA, such as full automation, cost-effectiveness, reproducibility, fast and precise measurement. Additionally, literature evidence shows that CLIA immunoassays have high diagnostic sensitivity and specificity for detecting anti-SARS-CoV-2 antibody levels [5, 15, 16].

In this study, we compared the analytical performance of three commercially available CLIA immunoassays for monitoring the immune response in a large cohort of vaccinated individuals after two and third BNT162b2 vaccine doses.

## Material and methods

### Study population

This observational retrospective study was performed at the University Hospital “P. Giaccone” of Palermo. All consecutive vaccinated subjects with age ≥ 18 years presenting to the Laboratory Medicine Unit of the University Hospital of Palermo from January to November 2021 to measure anti-S-RBD IgG levels were enrolled in the study. All subjects received two doses of the BNT162b2 vaccine (Pfizer-BioNTech) twenty-one days apart. A sub-group of subjects also received the third dose of the BNT162b2 vaccine. According to the government's guidelines for SARS-CoV-2 vaccination, the dosing interval was 3 weeks [18].

The study was conducted according to the guidelines of the Declaration of Helsinki and approved by the Institutional Review Board of the University Hospital of Palermo (nr 10, November 25, 2020). Informed consent was obtained from all individuals involved in the study.

For each subject, serum was obtained after centrifugation for 15 min at 4000xg at room temperature and aliquoted into three 500 μL aliquots before the immunochemical analyses. The aliquots of serum not immediately processed were stored at − 80 °C.

To further evaluate the analytical performances of the three methods, 40 serum samples by unvaccinated subjects without previous SARS-CoV-2 infection or other pathology were considered. Serum samples from the 40 subjects were collected before the COVID-19 pandemic and stored at − 80 °C until analysis. All serum samples were tested by the three assays.

### Anti-SARS-CoV2- antibody detection

We measured anti-SARS-CoV-2 antibody levels by three different CLIA assays, whose characteristics are summarized in Table 1.

**Table 1:** Analytical and technical features of evaluated anti-SARS-CoV-2 antibody immunoassays

Assay	Manufacturer	Assay principle	Analyzer	Antigen target	Cut-off
MAGLUMI Anti-SARS-CoV-2 S-RBD IgG	SNIBE	CLIA	Maglumi 800	RBD IgG	> 4.33 BAU/mL
Access SARS-CoV-2 IgG (1st IS)	Beckman Coulter	CLIA	Access 2	RBD IgG	> 30 IU/mL
Access SARS-CoV-2 IgG II	Beckman Coulter	CLIA	Access 2	RBD IgG	> 10 AU/mL

*AU* arbitrary units, *BAU* binding antibody units, *CLIA* chemiluminescent immunoassay, *Ig* immunoglobulin, *RBD* receptor-binding domain

#### Anti-S-RBD IgG antibody by MAGLUMI® SARS-CoV-2 S-RBD IgG

The MAGLUMI® SARS-CoV-2 S-RBD IgG (SNIBE-Shenzhen New Industries Biomedical Engineering Co., Ltd, Shenzhen, China) assay is designed for the quantitative detection of anti-S-RBD IgG antibodies.

The measurement was taken by indirect chemiluminescence immunoassay on Maglumi 800 (SNIBE-Shenzhen New Industries Biomedical Engineering Co., Ltd, Shenzhen, China) instrumentation, according to the manufacturer’s instructions. The assay has a limit of detection (LoD) of 0.7794 Binding Antibodies Units (BAU)/mL, as declared by the manufacturer. The unit of measurement used is in accordance with the latest notification received from World Health Organization (WHO) (Notice WHO Standard (20/136) Unit Conversion-RN21040201).

#### Access SARS-CoV-2 IgG (1st IS) measurement

The Access SARS-CoV-2 IgG (1st IS) (Beckman Coulter, Brea, CA, USA) assay is a paramagnetic particle, chemiluminescent immunoassay intended for the quantitative and qualitative detection of anti-S-RBD IgG antibody. The measurement was performed by indirect chemiluminescence immunoassay on Access 2 (Beckman Coulter) instrumentation, according to the manufacturer’s instructions. Access IU/mL is correlated with the First WHO International Standard Anti-SARS-CoV-2 Immunoglobulin (Human), NIBSC code, 20/136, in BAU/mL (BAU: binding antibody units).

To manually convert IU/mL concentrations to BAU/mL, multiply IU/mL by multiplication factor 1. The assay has a limit of detection (LoD) of 8 International Unit (IU)/ mL, as declared by the manufacturer. The analytical features of the assays used are shown in Table 1.

#### Access SARS-CoV-2 IgG II measurement

The Access SARS-CoV-2 IgG II (Beckman Coulter, Brea, CA, USA) assay is a paramagnetic particle, chemiluminescent immunoassay for the semi-quantitative and qualitative detection of anti-S-RBD IgG antibody. The measurement was performed by indirect chemiluminescence immunoassay on Access 2 (Beckman Coulter) instrumentation, according to the manufacturer’s instructions. The assay has a limit of detection (LoD) of ≤ 2.00 arbitrary unit (AU)/mL, as declared by the manufacturer.

### Statistical analysis

Statistical analyses were performed by SPSS statistical software v.17.0 (SPSS Inc., Chicago, IL, USA) and R Language v.4.0.3 (R Foundation for Statistical Computing, Vienna, Austria). Normality distribution was assessed by q–q plot and by the Shapiro–Wilk test. Quantitative variables were expressed by the median and interquartile range (IQR), while qualitative variables were expressed as absolute or relative frequencies. The correlation was evaluated by the nonparametric Spearman test, with the 95% confidence interval calculated by the bootstrap percentile method (5000 bootstrap replicates). Paired differences between groups were evaluated by the nonparametric Friedman test. Method comparison was evaluated by the nonparametric Passing–Bablok regression.

## Results

### Analytical performance of the three assays

Within laboratory repeatability was verified using 3 Quality Control (QC) levels for anti-S-RBD IgG (lev1: 2.0 BAU/mL, lev2: 17.8 BAU/mL; lev3: 68.6 BAU/mL), 2 QC levels for Access SARS-CoV-2 IgG (1st IS) (lev1: 0.27 IU/mL; lev2: 124.6 IU/mL) and 2 QC levels for Access SARS-CoV-2 IgG II (lev1: 0.07 AU/mL; lev2: 30.0 AU/mL). Repeatability, expressed as CV%, is reported in Table 2.

**Table 2:** Within-laboratory repeatability verified by quality controls (QC), reported as coefficient of variation (CV%)

	Analytical method (mean; CV%)		
QC	Anti-S-RBD IgG (BAU/mL)	SARS-CoV-2 IgG (1st IS)	SARS-CoV-2 IgG II (AU/mL)
Level 1	2.0; 22.8%	0.27; 20.4%	0.07; 21.6%
Level 2	17.8; 7.7%	124.6; 6.4%	30.0; 4.9%
Level 3	68.6; 7.1%		

Method performances were firstly verified in a cohort of 40 unvaccinated subjects without previous SARS-CoV-2 infection. IgG levels were transformed into a binary variable (negative *vs* positive) according to each method cut-off: < 4.33 versus ≥ 4.33 BAU/mL for anti-S-RBD IgG; < 30 versus ≥ 30 IU/mL for Access SARS-CoV-2 IgG (1st IS) and < 10 versus ≥ 10 AU/mL for Access SARS-CoV-2 IgG II. While all subjects were correctly classified as negative by Access SARS-CoV-2 IgG (1st IS) and Access SARS-CoV-2 IgG II, up to 5 subjects (12%) were erroneously identified as positive by anti-RBD IgG (although with Ig levels very close to the cut-off and ranging from 4.38 to 7.11 BAU/mL).

### Analytical performance of the three assays for evaluating vaccine-induced antibody levels

The anti-SARS-CoV2 antibody levels were measured in 600 vaccinated subjects, M:F 274:326 (46%:54%), with a median age of 55 years (IQR 40–65). Four hundred and eighty-seven (81.1%) subjects were measured only one time, whereas 90 (15.0%), 18 (3.0%), 4 (0.7%) and 1 (0.2%) displayed, respectively, 2, 3, 4 and 5 multiple antibody results, for a total of 742 measurements.

**Fig. 1:**
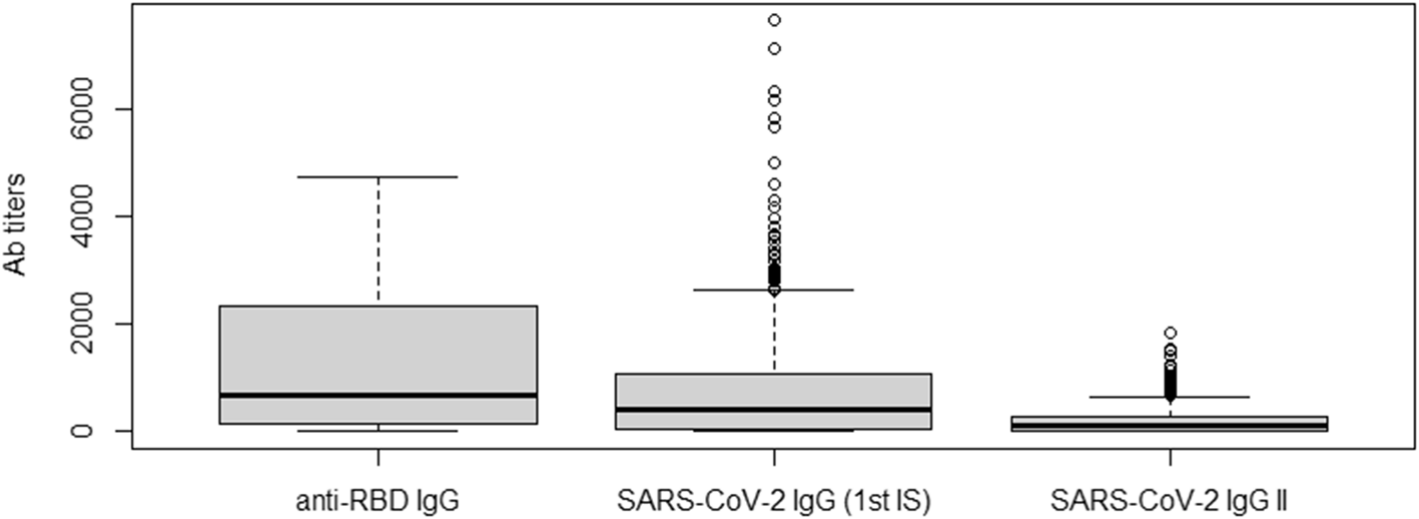
Anti-SARS antibody levels measured with MAGLUMI® SARS-CoV-2 S-RBD IgG, Access SARS-CoV-2 IgG (1st IS) and Access SARS-CoV-2 IgG II

When multiple measurements were available, only the first result of each patient was considered. Median (IQR) anti-S-RBD IgG, Access SARS-CoV-2 IgG (1st IS), Access SARS-CoV-2 IgG II levels of the 600 subjects investigated were, respectively, 687 BAU/mL (131–2325), 419 IU/mL (58–1091) and 104 AU/mL (14–274) (Fig. 1). Since samples with anti-S-RBD IgG levels higher than 4330 BAU/mL were not diluted, these samples were not considered for method comparison analysis and kinetics (included samples *N* = 517).

### Correlation analysis for the three assays

**Fig. 2:**
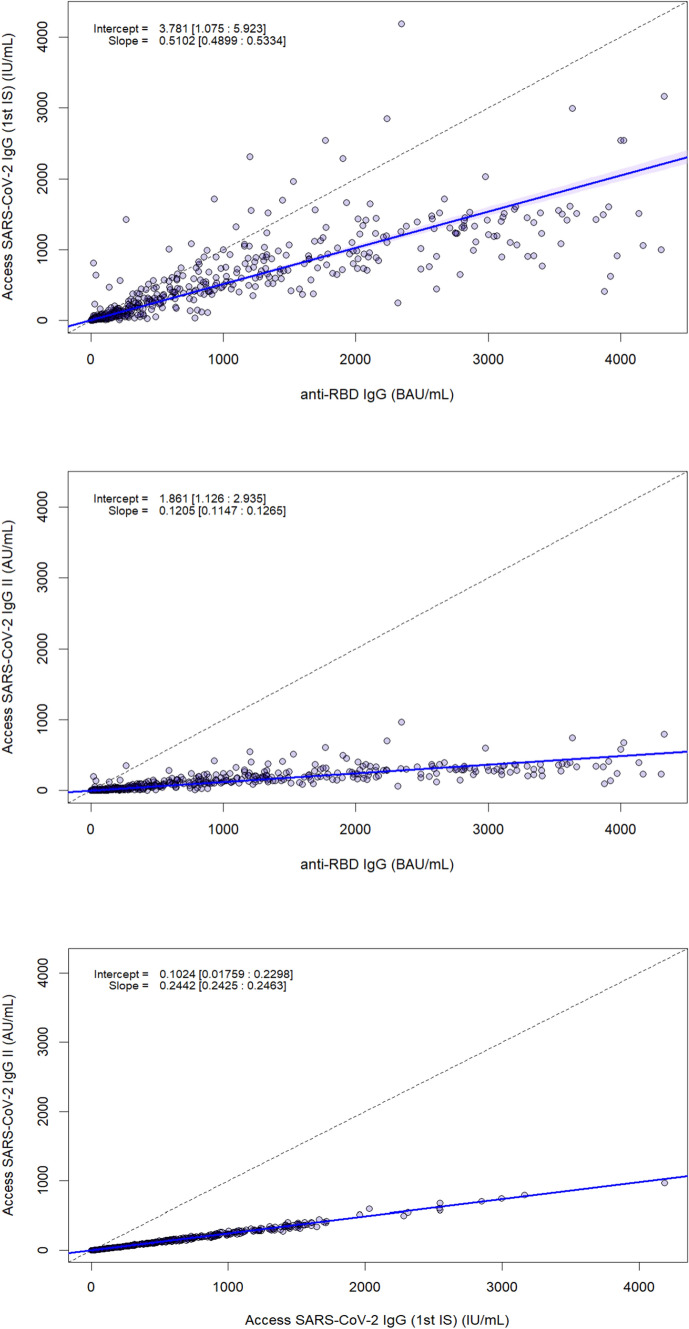
PB regression for MAGLUMI® SARS-CoV-2 S-RBD IgG, Access SARS-CoV-2 IgG (1st IS) and Access SARS-CoV-2 IgG II

Correlation between antibody levels, as assessed by nonparametric Spearman’s correlation, was rho = 0.930 (*p* < 0.001; 95%CI 0.908–0.946) for the pair anti-S-RBD IgG and Access SARS-CoV-2 IgG (1st IS), rho = 0.928 (*p* < 0.001; 95%CI 0.907–0.943) for the pair anti-S-RBD IgG and Access SARS-CoV-2 IgG II; rho = 0.999 (*p* < 0.001; 95%CI 0.998–0.999) for the pair Access SARS-CoV-2 IgG (1st IS) and SARS-CoV-2 IgG II (Table 3). At the Passing–Bablok regression, slopes and intercepts were, respectively, 0.51 (95%CI 0.49–0.53) and 3.78 (95%CI 1.07–5.92) for the pair anti-RBD IgG and Access SARS-CoV-2 IgG (1st IS) (Fig. 2), 0.12 (95%CI 0.11–0.13) and 1.86 (95%CI 1.13–2.93) for the pair anti-S-RBD IgG and Access SARS-CoV-2 IgG II (Fig. 2), 0.24 (95%CI 0.24–0.25) and 0.10 (95%CI 0.02–0.23) for the pair Access SARS-CoV-2 IgG (1st IS) and SARS-CoV-2 IgG II (Fig. 2).

**Table 3:** Spearman's correlation of SARS-COV-2 antibodies

	Access SARS-CoV-2 IgG (1st IS)	Access SARS-CoV-2 IgG II	MAGLUMI Anti-SARS-CoV-2 S-RBD IgG
Access SARS-CoV-2 IgG (1st IS)	–	0.999 (95%CI, 0.998–0.999) *p* < 0.001;	0.930 (95%CI, 0.908–0.946) *p* < 0.001;
Access SARS-CoV-2 IgG II	–	–	0.928 (95%CI, 0.907–0.943) *p* < 0.001;

*CI* confidence interval, *Ig* immunoglobulin, *RBD* receptor-binding domain, *S* spike protein

### Analytical performance of the three assays for evaluating kinetics of vaccine-induced antibody levels

Methods were also compared, considering antibody kinetics of the subjects with multiple measurements (*n* = 92). The median (IQR) time between the first and last measurements was 110 days (61–202). Since methods reported here were expressed in different units, kinetics were calculated as relative decreasing gradients ([[first measurement—last measurement]/[first measurement]]/[difference in days between first and last measurement] × 100%).

Median (IQR) percentage decreasing gradients per day were 0.65% (0.42–1.01), 0.70% (0.47–0.95) and 0.66% (0.47–0.97). Differences between percentage decreasing gradients were not statistically significant (overall Friedman test *p* = 0.415). Correlation between percentage decreasing gradients were, respectively, rho = 0.740 (*p* < 0.001; 95%CI 0.550–0.889) for the pair anti-S-RBD IgG and Access SARS-CoV-2 IgG (1st IS), rho = 0.758 (*p* < 0.001; 95%CI 0.577–0.898) for the pair anti-S-RBD IgG and Access SARS-CoV-2 IgG II; rho = 0.979 (*p* < 0.001; 95%CI 0.950–0.995) for the pair Access SARS-CoV-2 IgG (1st IS) and SARS-CoV-2 IgG II.

## Discussion

In this study, we compared the analytical performance of three commercially available immunoassays, all based on the CLIA method and detecting IgG antibodies against the spike glycoprotein RBD. The main finding of our study can be summarized as follows: i) the three assays investigated showed similar repeatability performances; ii) using a cohort of unvaccinated subjects without previous SARS-CoV-2 infection, optimal specificity was found for Access SARS-CoV-2 IgG (1st IS) and Access SARS-CoV-2 IgG II, whereas specificity was lower for anti-RBD IgG (although false positives displayed values very close to the cut-off). However, a more robust estimate of anti-RBD IgG specificity would require a higher sample size; iii) robust seropositive responses were observed for all three assays; iv) high correlation (for all comparisons rho > 0.928) between methods, although with non-overlapping titters (as demonstrated by significant regression slopes, and significant differences between methods); v) we observed overlapping decreasing kinetics of post-vaccination neutralizing antibodies by all methods tested. Thus, overall our findings showed that all assays have good analytical performance and a high correlation between them. However, as expected, the values of antibody levels vary greatly depending on the method used. This is in accordance with previous studies that compared the analytical performance of other immunoassays [19–21]. Although assays are based on the same analytical technique and detect the same antibodies both in terms of class and recognized epitopes, the results are different across methods in terms of single data [22, 23]. This can be relevant for monitoring a single patient, determining the adequate levels of antibodies as well as evaluating the permanence of immunity over time. Thus, to compare the magnitude of humoral response among different subjects and monitor it over time in the same subject, it should always be used the same assay.

A limitation of the study is that the absolute values of anti-SARS-CoV-2 antibodies were not compared, as only two of these provided results in BAU/mL according to the new WHO international standard 20/136. Moreover, we were aware of the infection status of the subjects included in the study.

Understanding the serological response to SARS-CoV-2 is essential to evaluate the impact of infection and disease globally, as well as for monitoring infected subjects and the vaccinated population over time. In clinical practice, it could represent a screening tool for immunosuppressed patients to assess prior vaccine response to new SARS-CoV-2 infection and to identify high-risk individuals eligible for monoclonal antibody combination therapy or community-provided prophylaxis.

Our data, obtained on a large cohort, suggest that although the immunoassays evaluated show similar analytical characteristics, their results cannot be compared. Thus, further efforts to standardize the analytical methods are mandatory.
